# Supplementary figures and images for: Stimulus-induced narrow-band gamma oscillations in humans can be recorded using open-hardware low-cost EEG amplifier
Source: PLoS One. 2023 Jan 23;18(1):e0279881. doi: 10.1371/journal.pone.0279881 (PMC9870151; doi:10.1371/journal.pone.0279881)

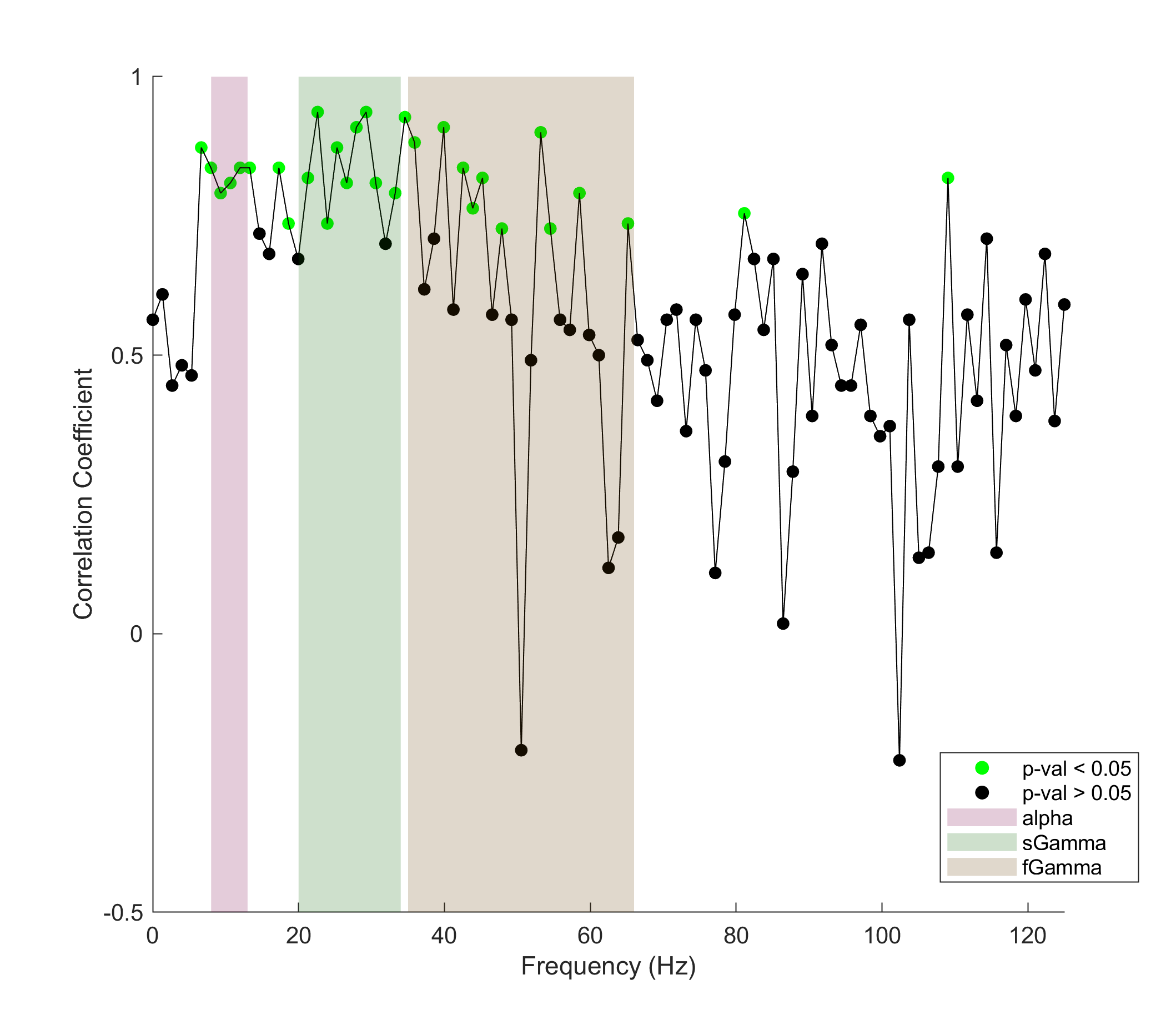

Supplement: S1 Fig — Black circles are for correlation values whose p-values calculated with permutation test are more than 0.05 and green circles are for correlation values whose p-values are less than 0.05. False Discovery Rate of p-values is controlled using Benjamini and Hochberg (1995) procedure. (TIF) [file pone.0279881.s001.tif]
